# Dataset on a 173 region awake resting state quantitative cerebral blood volume rat brain atlas and regional changes to cerebral blood volume under isoflurane anesthetization and CO_2_ challenge

**DOI:** 10.1016/j.dib.2018.01.021

**Published:** 2018-01-31

**Authors:** Codi A. Gharagouzloo, Liam Timms, Ju Qiao, Zihang Fang, Joseph Nneji, Aniket Pandya, Praveen Kulkarni, Anne L. van de Ven, Craig Ferris, Srinivas Sridhar

**Affiliations:** a Nanomedicine Science and Technology Center, Northeastern University, Boston, MA, United States; b Department of Bioengineering, Northeastern University, Boston, MA, United States; c Gordon Center for Medical Imaging, Massachusetts General Hospital, Harvard Medical School, Boston, MA, United States; d Department of Physics, Northeastern University, Boston, MA, United States; e Department of Mechanical and Industrial Engineering, Northeastern University, Boston, MA, United States; f Center for Translational NeuroImaging, Northeastern University, Boston, MA, United States; g Psychology Department, Northeastern University, Boston, MA, United States; h Department of Pharmaceutical Sciences, Northeastern University, Boston, MA, United States

## Abstract

The data in this article provide detail regarding the rat brain atlas measurements discussed in our research article, “Quantitative vascular neuroimaging of the rat brain using superparamagnetic nanoparticles: New insights on vascular organization and brain function” (Gharagouzloo et al., 2017) [1]. This article provides datasets of quantitative cerebral blood volume (qCBV) measurements across 173 regions of the rat brain in 11 healthy rats. State-changes from this baseline during isoflurane and CO_2_ administration are provided for all regions and all animals.

**Specifications Table**

**Table:** 

Subject area	*Biology*
More specific subject area	*Neurophysiology, vascular organization*
Type of data	*Cerebral Blood Volume*
How data was acquired	*QUTE-CE MRI*
Data format	*.xlsx, Processed per-animal CBV values for 173 regions derived from MRI data*
Experimental factors	*Animal was scanned with QUTE-CE MRI technique with and without contrast agent to allow for measurement of qCBV. Animal was scanned in awake, anesthetized by isoflurane and in hypercapnia from CO2 inhalation.*
Experimental features	*The baseline awake values and changes from these values during isoflurane and CO2 inhalation were determined.*
Data source location	*7 T MRI scanner at Northeastern University, Boston, MA*
Data accessibility	*Data is included with this article.*

**Value of the data**

• Provides measurements of the absolute cerebral blood volume (qCBV) in healthy awake adult male Sprague Dawley rats. This may be used as a comparison in alternative techniques, or for measuring changes away from this baseline in neuropathies or drug-induced states.
• Shows changes from the awake state to the anesthetized state (isoflurane). These changes may need to be considered in all studies using an anesthetized animal model.
• Provides a measure of vascular reactivity/perfusion reserve under CO_**2**_ challenge. This data provides a quantitative point of reference for other studies in various disease states.

## 1 Data

The data included here provides the average qCBV for the 11 rats across the 173 regions in the atlas in the awake state; averages and standard deviations for individual animals and all regions are also included (Data Set I). Also, included with this data set is the regional type categorization (1, 2 or 3) and the metric used to make this determination (Mean-Median Intensity Difference). Additionally, differences between anesthetized (Data Set II) and hypercapnic states (Data Set III) from this baseline are included. Along with these individual animal changes from the awake baseline, averages are derived, p-values and positive false discovery rate (pFDR) values are given for these differences. Circuits of note are also highlighted.

## 2 Experimental design, materials and methods

Methods are described in detail in our Research Article (Gharagouzloo, et al. [1]). The animal experiments were performed with 11 male Sprague Dawley weighing approximately 300 g each. They were conducted according to a protocol approved by the Northeastern University Division of Laboratory Animal Medicine and Institutional Animal Care and Use Committee. All MRI image acquisition, was completed on a Bruker Biospec 7.0 T/20 cm USR horizontal magnet (Bruker, Billerica, Massachusetts, USA) equipped with a 20-G/cm magnetic field gradient insert (ID = 12 cm, Bruker) and a custom built 30 mm diameter 300 MHz quadrature volume coil (Animal Imaging Research, Holden, MA). The coil included head restraints for awake animal imaging. Prior to image acquisition, rats were acclimated to the MRI scanning procedure and these restraints via the same method described in Ferris et al. [2].

In the experiments, the animals were initially anesthetized, secured in the scanner with a facemask continuing to deliver 1–3% isoflurane and QUTE-CE MRI images taken before contrast administration. The animals were given 25 min to recover while remaining in the scanner and a 6 mg/ml Fe dose of Ferumoxytol was administered via a tail vein catheter. After contrast agent injection, rats were exposed to 5% CO_2_ through the facemask and more images were acquired. The animals were then imaged while only air was administered through the mask. After this, 3% isoflurane was delivered through the facemask until the breathing rate fell to 30–40 breaths/min. The final QUTE-CE images were acquired during this state with continued isoflurane delivery.

Detail on the QUTE-CE MRI technique is provided in Gharagouzloo et al. [3]. Here, the parameters were time-to-echo (TE) = 13 µs, repetition-time (TR) = 4 ms, flip-angle (FA) = 20° and a field of view of 3 × 3 × 3 cm^3^ with 150 µm isotropic resolution. Two averages were used in creating the raw intensity image for each state. In the pre-processing, these raw images were corrected for B1-inhomogeneity, motion-corrected and aligned spatially. Blood MRI signal intensities were found for each individual scan and a 173-region atlas rat brain atlas was positioned on each aligned image set. Average MRI signal intensities were extracted per-region for each animal. The quantitative cerebral blood volume was calculated according to a partial-voluming theory. The raw MRI intensities were also used to characterize regional types: the difference in the mean and median of the raw intensity per-voxel per-region provided a measure of skewness.

Differences from the awake air breathing baseline state to the CO_2_ challenged and isoflurane exposed states were calculated for each individual animal across all 173 regions. The set of differences for each region was tested against a null hypothesis of zero change with a one-sample *t*-test. Each circuit-level analysis was completed by combining raw per-voxel intensity data from the relevant regions and analyzing it according to the same procedure; i.e. treating each circuit as a single larger region in addition to its components. Imaging data, including the values presented here mapped to the 3D Atlas, are presented in Gharagouzloo et al. [1].
