# Supplementary material for: Dataset on a 173 region awake resting state quantitative cerebral blood volume rat brain atlas and regional changes to cerebral blood volume under isoflurane anesthetization and CO2 challenge
Source: Data Brief. 2018 Jan 31;17:393–6. doi: 10.1016/j.dib.2018.01.021 (PMC5988288; doi:10.1016/j.dib.2018.01.021)
Supplement: Supplementary file 1 — Transparency document [file mmc1.docx]

QUTE-CE MRI is the subject of a patent application assigned to Northeastern University (inventors Codi A. Gharagouzloo and Srinivas Sridhar).
